# Identification of fish species through tRNA-based primer design

**DOI:** 10.1186/s12859-022-04717-8

**Published:** 2022-12-06

**Authors:** Ting-Hui Wu, Cing-Han Yang, Tun-Wen Pai, Li-Ping Ho, Jen-Leih Wu, Hsin-Yiu Chou

**Affiliations:** 1 Department of Computer Science and Engineering, National Taiwan Ocean University, Keelung, Taiwan; 2 Department of Computer Science and Information Engineering, National Taipei University of Technology, Taipei, Taiwan; 3 Department of Aquaculture, National Penghu University of Science and Technology, Penghu, Taiwan; 4 Department of Bioscience and Biotechnology, National Taiwan Ocean University, Keelung, Taiwan; 5 Institute of Cellular and Organismic Biology, Academia Sinica, Taipei, Taiwan; 6 Department of Aquaculture, College of Life Science, National Taiwan Ocean University, Keelung, Taiwan

**Keywords:** Fish barcode, Mitochondrial, tRNA gene, Primer design

## Abstract

**Background:**

The correct establishment of the barcode classification system for fish can facilitate biotaxonomists to distinguish fish species, and it can help the government to verify the authenticity of the ingredients of fish products or identify unknown fish related samples. The Cytochrome c oxidation I (COI) gene sequence in the mitochondria of each species possesses unique characteristics, which has been widely used as barcodes in identifying species in recent years. Instead of using COI gene sequences for primer design, flanking tRNA segments of COI genes from 2618 complete fish mitochondrial genomes were analyzed to discover suitable primers for fish classification at taxonomic family level. The minimal number of primer sets is designed to effectively distinguish various clustered groups of fish species for identification applications. Sequence alignment analysis and cross tRNA segment comparisons were applied to check and ensure the primers for each cluster group are exclusive.

**Results:**

Two approaches were applied to improve primer design and re-cluster fish species. The results have shown that exclusive primers for 2618 fish species were successfully discovered through in silico analysis. In addition, we applied sequence alignment analysis to confirm that each pair of primers can successfully identify all collected fish species at the taxonomic family levels.

**Conclusions:**

This study provided a practical strategy to discover unique primers for each fishery species and a comprehensive list of exclusive primers for extracting COI barcode sequences of all known fishery species. Various applications of verification of fish products or identification of unknown fish species could be effectively achieved.

**Supplementary Information:**

The online version contains supplementary material available at 10.1186/s12859-022-04717-8.

## Background

Seafood products have been tremendously increased globally due to they are considered as prime source of high quality protein. There are more than 32,500 species of fishes exist worldwide [1], and among them, a lot of economic fishes are processed as fillets and minced flesh instead of whole fish to be shipped around the world. How to provide appropriate identification techniques for reliable and accurate information of fish products becomes a challenge task. In addition to the issues of responsible trade for fish products, several unseen fish species are continually discovered in developed countries and these new fishes could only be identified by few taxonomists. Due to insufficient taxonomic expertise training in most developed countries, novel and promising techniques for fish identification should be proposed to solve this dilemma [2].

There are several different approaches for fish identification approaches, which can be categorized by using whole fish or part of fish body to identify fishes. For using whole fish organism includes expert authority (taxonomists and local experts), image and specimen only (local reference collections and image recognition systems), identification keys (dichotomous keys, interactive electronic keys, morphometrics); for parts of fish body includes anatomy (scale and otoliths) and genetic sequences (single nucleotide polymorphisms and barcode). The introduction and corresponding criteria of these different identification approaches can be found and compared in [2].

A major advantage of adopting genetic sequence based analytical procedures to identify fishes is that the approach is especially preferable and practical for food supply chain issues. It can be done by taking from whole specimens to a small portion of fish (scales or fins), and it also works for highly processed fish products. Compare to depending on taxonomic experts, the use of DNA-based approach for identification can reduce the required time and increase accuracies for inexperienced taxonomists. The idea of DNA barcoding was first proposed by Paul Hebert, a Canadian zoologist [3]. The concept of DNA barcoding is like the tangible barcodes commonly seen in daily life. Compared to unified traditional barcode images, the DNA barcode represents specific genetic DNA sequences of a specimen. The double helix structure of a DNA sequence is formed from four deoxyribonucleotides, namely A, T, C, and G, and contains genetic information that is unique to each species. This property is similar to the barcode images that are uniquely represented for different products. Hence, DNA barcode sequence data can be used for species identification [4–6].

DNA barcoding technology requires a short DNA sequence that is unique across species and highly conserved among the same species. Since 2003, many studies have revealed that throughout the process of biological evolution, one segment of the mitochondrial gene sequence called the cytochrome C oxidase I (COI) gene has been highly conserved among the same species and highly unique across species [7]. Highly conserved indicates that the COI gene sequences of the same species have high similarity. In other words, the COI gene sequences of the same species do not vary significantly throughout the process of evolution. From previous study of Australia’s 207 species of fish, the average distances or dis-similarities of COI genes within-species, genus, family, order and class increased from 0.39 to 23.27% [8]. The evolutionary distance measurement indicates that the COI gene sequences of different biological classification levels are highly variable. In other words, although the COI gene sequence of the same species may not be identical after evolution, the sequence similarity between the same species is higher than that of other species. Therefore, high conservation of COI gene sequences among the same species and uniqueness among different species make the COI gene sequences eligible for DNA barcoding system. By comparing multiple COI gene sequences, we can distinguish the genetic relationship among species through sequence similarity analysis.

DNA barcode validation requires replication of target sequences using PCR amplification techniques. Up to 98% of species successfully use PCR technology for DNA barcoding, but for a small number of species, the success rate of PCR technology is very low. Since this problem hampers the progress of research, many researchers have developed new primers to increase the success rate of COI gene sequence barcoding using PCR technology [9]. In the study "Recovery of the viable COI barcode region in diverse Hexapoda through tRNA-based primers" [10], most of the primer designs of arthropods were based on the tRNA (transfer RNA) gene sequence located upstream of the COI gene sequence. These tRNAs are a type of ribonucleic acid composed of 76–90 nucleotides. Various biologically diverse species contain short conserved sequences among the same species that can be used to design primers. Therefore, we used the tRNA gene sequences located upstream and downstream of the COI gene to design and develop new primers for effective identification of fish species.

It can be observed that fish related products are important to human food from statistical reports. Fishery products provided more than 17 percent of total animal protein and 7 percent of all proteins for human [11]. Hence, it can be expected that when a specific fish species could be accurately identified through designed unique tRNA-based primers and applied them to extract corresponding COI barcode sequences for target species validation, and various applications of verification of fish products or identification of unknown fish species would be effectively achieved. Accordingly, to accurately identify fish related products becomes necessary not only in security issues, but also in monitoring fisheries for long term sustainability in terms of biodiversity conservation and ecosystem research. This study could provide a novel and practical strategy to discover unique primers for each fishery species and a comprehensive list of exclusive primers for extracting COI barcode sequences of all known fishery species.

## Results and discussion

### Multiple sequence alignment analysis

The 2618 retrieved mitochondrial genomes were classified into 397 families according to the NCBI taxonomy, which contained 160 single-species and 237 multi-species groups. Multiple sequence alignment tools were used to calculate average similarity scores of the paired gene sequences within the 237 multispecies group. The average aligned score for the L-tRNA gene sequences is 976, COI gene sequences 949, and R-tRNA gene sequences 989. The score for two identical sequences is 1000. A higher alignment score represents higher similarity of aligned sequences. In addition, both Clustal Omega and T-Coffee were used to calculate the average genetic distance between species within a group. The species possessing a highest average similarity value within a clustered group was selected as the representative species of the group, and it was used to calculate the sequence similarity scores of the COI genes and primers against all other representative sequences among the 397 groups. The results showed that an average similarity score of L-tRNA gene segments was 801, COI gene sequence 774, and R-tRNA 910, as shown in Table 1. The average score of the primers between the groups indicated high similarity.

**Table 1:** The average similarity scores of the gene sequences

T-Coffee score^1^	L-tRNA	COI	R-tRNA
Intra-species average similarity	976	949	989
Inter-species average similarity	801	774	910

^1^700 ≤ Score ≤ 1000 indicates high similarity; 400 ≤ Score < 700 indicates moderate similarity; 0 ≤ Score < 400 indicates low similarity

### Results for first stage of primer selection

It was checked whether both L-primer and R-primer could be designed simultaneously. Unfortunately, the results showed that 11 out of 2618 species lacked one strand for the primer design, and the 11 species were listed in Table 2. These 11 species belong to 8 family groups, and the strategy was changed to extend one more tRNA segment located upstream and downstream of the COI gene, and redesigned the primers.

**Table 2:** List of the 11 species with incomplete primer design

Family ID	Species ID	Species name
7746 (*Geotriidae*)	NC_029404	*Geotria australis*
7762 (*Myxinidae*)	NC_002807	*Eptatretus burgeri*
7869 (*Chimaeridae*)	NC_003136	*Chimaera monstrosa*
7869 (*Chimaeridae*)	NC_014288	*Chimaera fulva*
7869 (*Chimaeridae*)	NC_014290	*Hydrolagus lemures*
7944 (*Muraenesocidae*)	NC_013617	*Cynoponticus ferox*
8065 (*Batrachoididae*)	NC_006920	*Porichthys myriaster*
30761 (*Macrouridae*)	NC_027436	*Cetonurus globiceps*
31031 (*Tetraodontidae*)	NC_015368	*Colomesus asellus*
31031 (*Tetraodontidae*)	NC_015370	*Colomesus psittacus*
42148 (*Moronidae*)	NC_030281	*Morone Americana*

Each single-species group contained a pair of primers. After the minimum number of primers were obtained for multispecies groups, the designed primers were analyzed by Bowtie2 to determine whether each pair of primers held exclusive properties.

According to the NCBI Taxonomy, the 397 taxonomic groups were classified at the “family” level, among them, 160 groups contain single species and 237 groups contain multiple species within a group. In this study, exclusive primers represent that the designed primer pairs could be uniquely found for a specific family group, while the non-exclusive primers represent that the designed primer pairs could be crossly matched with primers of other fishery species. Since the primer designing core algorithms provide several candidate primers, various combinations of forward and reverse primers were formulated for cross comparison among all collected fishery groups. From the first stage primer selection and comparison, there are only 10 among 160 single-species groups contained non-exclusive single primer pairs. For the rest 237 multispecies groups: 56 groups contained exclusive single primer pairs, 157 groups contained exclusive multiple primer pairs, and 24 groups contained both exclusive multiple primer pairs and non-exclusive multiple primer pairs (Table 3). Furthermore, these 157 multispecies groups of fish contained 663 exclusive pairs of primers that can be accurately matched with 1,507 fish species, while the 24 groups contained 189 exclusive pairs of primers that can be accurately matched with 720 fish species, and the remaining 66 fish species were unable to find any exclusive primers and primer design for these fish species were compared to other species.

**Table 3:** Statistics of the exclusive and non-exclusive primers

	Groups (G)	Number of groups with exclusive primer pairs (EP)	Number of groups with (partial) non-exclusive primer pairs (NEP)
*Single-species group (SSG)*			
Single primer pairs (SP)	160	150	10
*Multispecies group (MSG)*			
SP	56	56	0
Multiple primer pairs (MP)	181	157	24

### Hypothetical reasons for the non-exclusive primers

Based on the results for primer design and sequence alignment, 76 fish species failed to find an exclusive primer. This experiment speculated the following two reasons. We checked two fish species without exclusive primers and found that the tRNA gene sequences, both upstream and downstream, differed by only one single base. This resulted in highly similar primer sequences and hence failed to be exclusive between these two groups. This experiment assumes that this result is one of the reasons for the failure to find an exclusive primer. The sequence contents are shown in Tables 4 and 5.

**Table 4:** The upstream forward-tRNA gene sequences of two single-species groups

Species	L-tRNA
*Auchenoglanis occidentalis*	
NC_015809	TGATAGGAAAAGGA**C**TTAAACCTTTGTTCATGGAGCTACAATCCACCGCCTAACCCTCGGCCATCCTACC
*Synodontis schoutedeni*	
NC_015808	TGATAGGAAAAGGA**T**TTAAACCTTTGTTCATGGAGCTACAATCCACCGCCTAACCCTCGGCCATCCTACC

**Table 5:** The downstream forward-tRNA gene sequences of two single-species groups

Species	R- tRNA
*Auchenoglanis occidentalis*	
NC_015809	CGAGAAAGGAAGGAATCGAACCCCCATAAACTAGTTTCAAGCCAGTCACATAACC**G**CTCTGTCACTTTCTT
*Synodontis schoutedeni*	
NC_015808	CGAGAAAGGAAGGAATCGAACCCCCATAAACTAGTTTCAAGCCAGTCACATAACC**A**CTCTGTCACTTTCTT

As shown in the illustrated example, due to the high similarity between the nearest tRNA gene sequences located in upstream or downstream of the COI gene, the next ordinarily tRNA genes located upstream of COI (i.e. the second nearest tRNA genes) were retrieved for the 10 single-species groups and the primers were redesigned according to the second tRNA genes on both upstream and downstream regions. Bowtie2 sequence mapping analysis showed that the number of groups with non-exclusive primers could be reduced from 10 to 2 groups by increasing one more neighboring tRNA segments that could be retrieved.

After extending an additional tRNA segment, there are yet two family groups remained with no exclusive primers. For these two single-species groups possessing no exclusive primers, we applied Bowtie2 to analyze the sequence alignment of the first five predicted candidate primer pairs of each species obtained from the primer design tool (Primer 3) instead of using only one predicted primer. All groups closely related to the current species were performed for sequence alignment. According to these two single-species group containing no exclusive primers, all related groups were joined to verify average similarity of COI sequences by T-Coffee and the results were shown in Table 6. However, there were no significant differences between the scores due to combining the different groups. The results were consistent with the previous report of phylogenetic classification of bony fish based on molecular phylogenies research [12]. For example, the *Zenarchopteridae* and *Belonidae* were classified as suborder of *Belonoidei* with relative high sequence similarities, while the family of *Lotidae* was no longer recognized as a single-species, and three genera (*Brosme*, *Lota*, and *Molva*) formerly in *Lotidae* are now included in *Gadidae*.

**Table 6:** Relationship between the two single-species groups and related groups

Group ID (Family ID)	Related group ID (Family ID)	T-coffee score	
		Average score before combing the related groups	Average score after combining the related groups
1489918 (*Zenarchopteridae*)	94935 (*Belonidae*)	949	952
81641 (*Lotidae*)	8045 (*Gadidae*)	974	968

### Optimization and statistical analysis of groups with exclusive and non-exclusive primers

According to the two phenomenons explained at the first stage, based on the first observation, two tRNA gene segments located upstream and downstream of COI gene sequences could be retrieved from the 8 groups, and based on the second observation, there are 29 groups with non-exclusive primers after combining the current and related groups, as shown in Table 7.

**Table 7:** Number of groups after optimization

	Reason one	Reason two
SSGSP^1^	6	4
MSGSP^2^	1	1
MSGMP^3^	1	24

^1^SSGSP: Single-Species Group Single Primer Pairs

^2^MSGSP: Multispecies Group Single Primer Pairs

^3^MSGMP: Multispecies Group Multiple Primer Pairs

According to the re-grouping processes, the number of single-species groups with single primer pairs decreased from 160 to 156, multispecies groups with single primer pairs were still at 56, and multispecies groups with multiple primers pairs reduced from 181 to 156. After optimization, 11 new groups were generated. The optimized values based on the two observations are shown in Tables 8 and 9.

**Table 8:** Optimized results for the first observation

	Number of groups with EP^4^	Number of groups with (partial) NEP^5^
SSGSP^1^	150 + 6 = 156	10 − 6 = 4
MSGSP^2^	56 + 1 = 57	0
MSPMP^3^	157 + 1 = 158	24 − 2 = 22

^1^SSGSP: Single-Species Group Single Primer Pairs

^2^MSGSP: Multispecies Group Single Primer Pairs

^3^MSGMP: Multispecies Group Multiple Primer Pairs

^4^EP: Exclusive Primer Pairs

^5^NEP: Non-exclusive Primer Pairs

**Table 9:** Optimized results for the second observation

	Number of groups with EP^4^	Number of groups with (partial) NEP^5^
SSGSP^1^	156	4 − 4 = 0
MSGSP^2^	57 − 1 = 56	0
MSPMP^3^	158 − 3 = 155	22 − 21 = 1

^1^SSGSP: Single-Species Group Single Primer Pairs

^2^MSGSP: Multispecies Group Single Primer Pairs

^3^MSGMP: Multispecies Group Multiple Primer Pairs

^4^EP: Exclusive Primer Pairs

^5^NEP: Non-exclusive Primer Pairs

Among the 397 groups, 156 single-species groups with single primer pairs and 56 multispecies groups with single primer pairs were found to be exclusive. However, among the 156 multispecies groups with multiple primer pairs, there was a group with non-exclusive primers. The 11 optimized multi-family groups contained 1 group with exclusive single primer pairs and 10 groups with exclusive multiple primer pairs. As shown in Table 10. When compared with the results of primer selection and primer sequence alignment from stage one, 160 single-species groups were reduced to 156 groups, 237 multispecies groups were reduced to 212 multispecies groups and 11 new multi-family groups. By analyzing the sequence alignment results of 397 groups, 156 pairs of exclusive primers from the single-species groups accurately matched with 156 fish species. Further, 212 multispecies groups contained 56 multispecies groups with single primer pairs and 156 multispecies groups with multiple primer pairs. In 56 multispecies groups with single primer pairs, 56 pairs of primers accurately matched with 167 fish species. In 156 multispecies groups with multiple primer pairs, including 155 groups that contain exclusive primer pairs, and 1 group contained both exclusive primer pairs and non-exclusive primer pairs. Furthermore, these 155 groups that contain 650 exclusive pairs of primers, accurately matched with 1,473 fish species, 1 group that contained 111 exclusive pairs of primers accurately matched with 583 fish species, and the remaining 5 fish species were unable to find any exclusive primers for these fish when compared to other species. According to the second observation, 11 multi-family groups, including 1 exclusive single primer pair accurately matched with 12 fish species and 10 exclusive multiple primer pairs accurately matched with 222 fish species.

**Table 10:** Statistics of groups with exclusive and non-exclusive primers after optimization

	Groups (G)	Number of groups with exclusive primer pairs (EP)	Number of groups with (partial) non-exclusive primer pairs (NEP)
*Single-species group (SSG)*			
Single primer pairs (SP)	156	156	0
*Multispecies group (MSG)*			
SP	56	56	0
Multiple primer pairs (MP)	156	155	1
Multi-Family Group (MFG)			
SP	1	1	0
MP	10	10	0

### Results for the second stage of primer selection

After the first stage of primer selection and primer sequence alignment, the primer design result of 5 fish species in the group belonging to the family ID 7953 did not match with any species (Table 11). The probable reason could be the large number of species in the group that gave rise to duplicate alignments of primers with other species. Therefore, these five species were selected for the second stage and their mitogenomic sequences were used as the reference sequences for sequence alignment analysis. The second stage contained two exclusive pairs of primers (Table 12).

**Table 11:** Non-exclusive primer species from the first stage

Family ID	Species ID	Species name
7953	NC_019575	*Alburnus tarichi*
	NC_022718	*Oxygymnocypris stewartii*
	NC_024880	*Schizopygopsis malacanthus*
	NC_024588	*Pseudogyrinocheilus prochilus*
	NC_036349	*Gymnocypris scleracanthus*

**Table 12:** The second stage contained two exclusive pairs of primers

Species ID	F-primer	R-primer
NC_019575	GCGTCTCTGGATTTGCAATCC	ACATGGGGGTTCAATTCCTCC
NC_022718 NC_024880 NC_024588 NC_036349	CTCTGTCTTCGGGGCTACAAC	GGGGGTTCAATTCCTCCCTTT

### Average similarity scores within and between groups

Among the 397 groups, the number of groups with exclusive primers was 378 and that with partially non-exclusive primers remained one group. In order to discuss the sequence similarity among designed primers and the screening methods within and between groups, we analyzed the sequence similarity of primers within 166 multispecies groups containing multiple primer pairs and obtained an average F-primer score of 692 and R-primer of 743. The average score of the primers within the groups indicated high similarities. In addition, Cluster Omega was used to calculate the average genetic distance between the species in the group. The species with the highest similarity value was selected as the representative species of the group and the average similarity score of the primers between 397 groups was calculated. The results of the F-primer score and the R-primer score were 209 and 358, respectively. The average score of the primers between the groups indicated low similarity.

### Fish barcode system

**Fig. 1:**
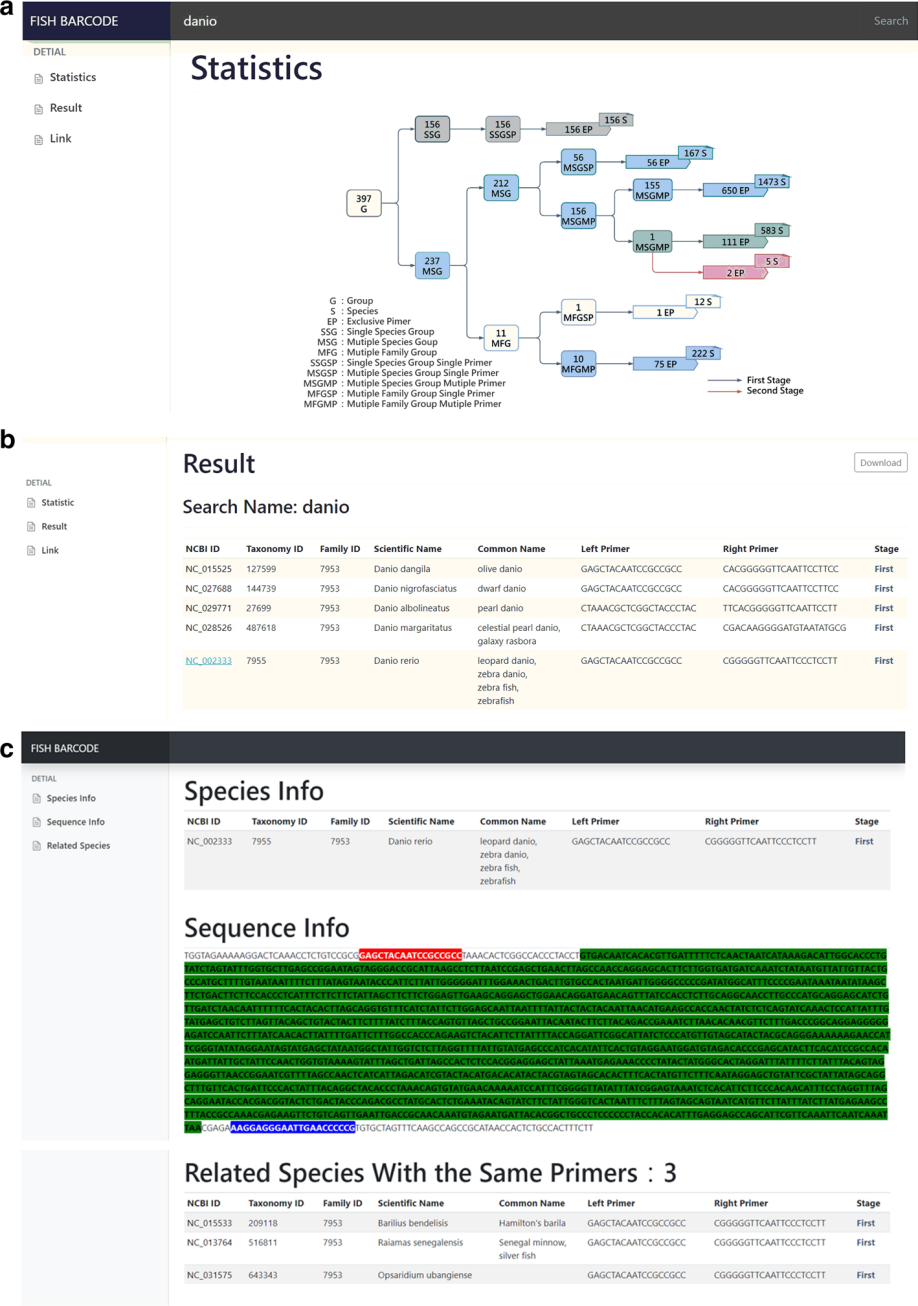
Fish barcode system: **a** home page interface; **b** an example of searched results for keyword of “danio”; **c** a resulting page for species information, sequence information and related species information of "Danio rerio"

This study presents the primer sequence information of 2618 fish species using standard web tools. The system requires a user to input the scientific name or common name of the species to be inquired through the web browser. After fuzzy query with keyword of the input name, the species information and the position between primers and COI gene sequence will be presented on the website, and all related species with the same primer pair as the search species were further provided on the website too. The home page shows the number of primer distribution and primer selecting stage of single-species groups and multi-species groups, and provides input fields on the top for users to input the name of species, as shown in Fig. 1a. All corresponding primer sequences can be found within the additional supporting file (Additional file 1).

For example, when a user providing a query word of "Danio", the system found 5 fish species related to the query keyword and displayed in the "Result" section, as shown in Fig. 1b. Users could click on the species in the "NCBI ID" column to view further information.

Taking "Danio rerio" as an example. After clicking on "NC_002333" species in NCBI ID field, the information of NCBI ID, taxonomic ID, family ID, scientific name, common name, left primer, right primer and stage about this species will be displayed in the list under "Species info". "Sequence info" showed the loci of left and right primer sequences and corresponding COI gene sequences. Red background represents left primer, blue for right primer and green for COI gene sequence. "Related Species with the Same Primers" shows three species information with the same primers as *Danio rerio*, as shown in Fig. 1c.

## Conclusion

In this study, 2618 sequences were classified into 397 families according to the NCBI taxonomy. T-Coffee was used to calculate the average similarity scores of the sequences within and between groups. The average scores of L-tRNA and R-tRNA indicated high similarity among the sequences within taxonomic family level, which suggested that the primer design based on the first flanking tRNA segments of COI gene could not accurately cluster fish species according to the classification rules provided by NCBI. To find designed primer pairs that are highly conserved within a family group and highly unique cross different family groups, extended tRNA segments located on upstream and downstream franking regions of the COI genes should be applied. However, due to limited number of tRNA segments in the mitochondria, if we continue to increase the number of tRNA segments, the distance between tRNAs and COI gene would lead to undesirable variations. Thus, unlimited extension of tRNAs was not considered as the best solution. An alternative solution to discover suitable primer pairs for distinguishing different species is to apply various combinations of L-tRNA and R-tRNA primer candidates from Primer3 tool. In this study, we used the first five pairs of the designed primers of each species to perform Bowtie2 and analyze the sequence alignment results. The group most closely related to the current species was calculated. Then, the T-Coffee multiple sequence alignment tool was used to calculate the average similarity score of the COI gene sequence before and after re-clustering the related group. We found that there were no significant differences between the similarity scores. Thus, this analytical results demonstrated that a few fish species with high similarities of COI genes could be clustered within the identical family cluster to reduce the problem searching exclusive primer pairs.

After the first trial of extending tRNA segments for primer selection, there were five fish species remained unable to be distinguished from other groups. This was because of the large number of species in the group that gave rise to duplicate alignments of primers with other species. Therefore, to further improve the distinguishable primer design, the mitogenomic sequences of the five fish species were used as the reference sequences for sequence mapping. Hence, according to the proposed two-stage exclusive primer design, the system could successfully cluster all 2618 fish species at the taxonomic family level.

Lastly, the average sequence-similarity scores of the selected primers were analyzed. The average similarity scores of the primers within the same groups indicated high similarity, while the low average s similarity core of the primers between the groups indicated attribute of exclusiveness. This suggested that the selection of the primers conforms to the family group with high conservation and homogeneity. Thus, based on the above results, we demonstrated a method, which was different from the traditional approach of using physical traits as classification standards. High sequence similarity among COI genes and associated primer sequences could be considered as a good criterion to validate fish species classification.

## Materials and methods

### Database of fish mitogenomes

The Mitofish (Mitochondrial Genome Database of Fish) is a collection of mitochondrial genomes. The database collects complete fishery mitochondrial genomes (2618 fish species) as well as certain partial gene sequences (553,044 sequences from 29,316 fish species) [13]. In this paper, mitogenomic data of 2618 fish species retrieved from the Mitofish database were used as the major data contents for analysis.

### Experimental procedures

Mitogenomic data for 2618 species of fish were downloaded from the Mitofish database. According to the annotation position and location obtained from MitoAnnotator, tRNA gene sequences located on both upstream and downstream of the COI gene sequence were retrieved and grouped at the “family” level according to the Taxonomy classification rules provided by NCBI [14]. The average pair sequence similarity within each family group was calculated and checked if they possess highly sequence similarities between species within an identical group and low sequence similarities between species in different groups.

**Fig. 2:**
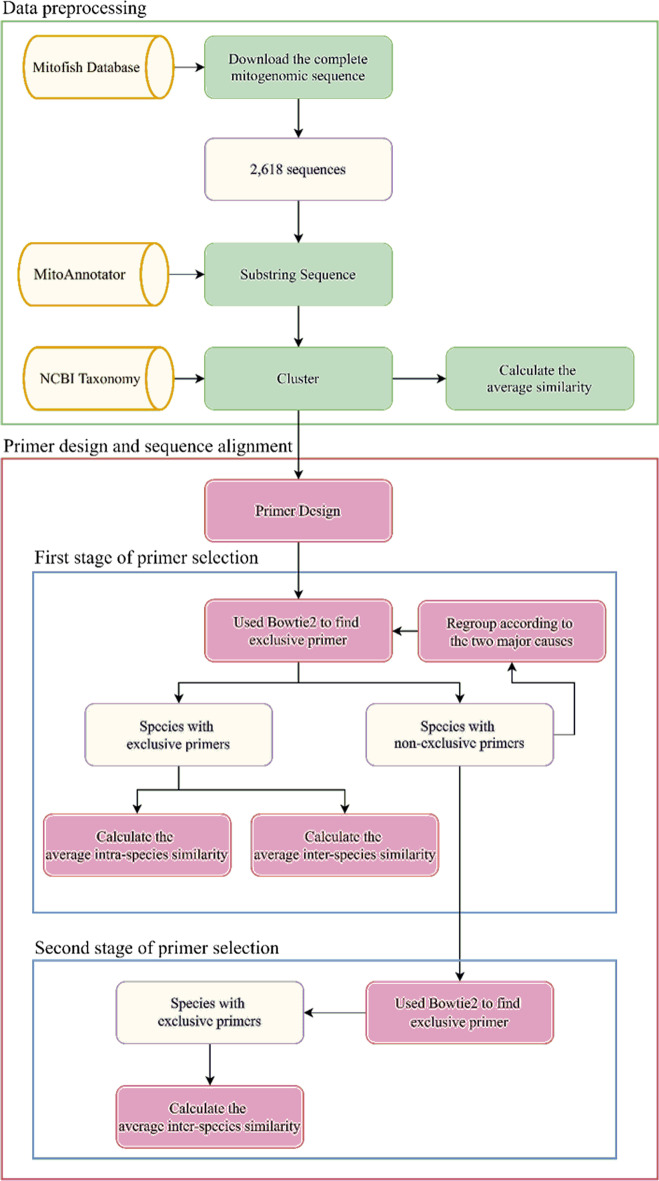
Schematic representation of the proposed two-stage processes

The primers of COI genes of 2618 fish species were analyzed using Primer3 and species with identical primers were merged according to the results obtained from the primer design. Primer selection was divided into two stages. At the first stage, the collected 2618 fish mitogenomes were used as reference sequences for primer sequence mapping analysis. Bowtie2 was applied to verify exclusive primers [15]. Based on the results of sequence alignment analysis, we hypothesized two possible reasons for explaining the clustered species groups without holding exclusive primers for each group: high sequence similarities within a clustered group and misclassification. To overcome these problems, a two-stage system for exclusive primer design is proposed. If non-exclusive primers could be identified for the first trial of primer design, the clustered groups could be isolated, and the groups without exclusive primers would be analyzed by extending franking segments for the second trial primer design. If exclusive primers could not be identified, a re-grouping process according to sequence similarity analysis would be proceeded and corresponding primers would be identified again for cross group comparison. The primer analytical module will be performed at the second stage of primer selection analysis and repeat sequence alignment procedures again to check whether the primers belonging to the same group and holding exclusive characteristics. Both T-Coffee (Tree based Consistency Objective Function For alignment Evaluation) (database version 12.00.7fb08c2) [16] and Clustal Omega [17] tools were used to calculate the sequence similarities between primers for various fishery groups. Figure 2 shows the schematic representation of the experimental processes.

### Multiple sequence alignment

In this procedure, T-coffee was used to calculate the average similarities among sequences within a group through a progressive multiple sequence alignment approach. It generated a library of pairwise alignments to guide the multiple sequence alignment.

1 $${\text{SCORE}} = \mathop \sum \limits_{i = 1}^{N - 1} \mathop \sum \limits_{j = i + 1}^{N} W_{i,j} \times COST\left( {A_{i,j} } \right)$$

where *N* represents the total number of sequences, *W*_*i,j*_ represents the weight of the sequence *i* and sequence *j*, and *COST*(*A*_*i,j*_) represents the transposition matrix for calculating penalty.

### Primer design

Primer3 (version 2.4.0) was used to design primers in this study [18]. One tRNA gene segment located at both upstream and downstream of the COI gene sequence was retrieved respectively that constituted a pair of primers. The forward-primer (F-primer) was located in the upstream tRNA gene sequence (L-tRNA) and the reverse-primer (R-primer) was located in the downstream tRNA gene sequence (R-tRNA). The final target sequence is the COI gene located between the F-primer and the R-primer. The length of primers on both sides can be set up to 26 bases at most, 18 bases at least, with an optimum length of 21 bases.

### Sequence alignment analysis of primers

In this study, Bowtie2 (version 2.3.4.1) was used to map sequences and analyze exclusive properties of Primer 3 designed primers for each group. The first candidate primer predicted by Primer 3 for a single-species group (a clustered group only contains single species) was initially selected as representative primers for the single-species group, and the Bowtie2 was applied to validate the uniqueness property of the selected primer. If the first predicted candidate primer was not uniquely occurred in to the corresponding group, the second predicted candidate primer by Primer 3 would be selected and identical sequence mapping analysis was performed. When a group containing multispecies, a conserved representative primer pair would be considered for all species in the same group. It can be expected that more than one pair of candidate primers occurred within a multispecies group, and sequence alignment analysis would be performed to validate conserved property of candidate primers within a group and uniqueness features among different groups. In order to analyze uniqueness of primers among different groups, Clustal Omega (version 1.2.4) was applied to calculate the genetic distance between species in a group, and it was used as a tool to select representative primer sequences.

## Supplementary Information

. **Additional file 1.** Fish Barcode.

## Abbreviations

COI: Cytochrome c oxidation I
tRNA: Transfer ribonucleic acid
Mitofish: Mitochondrial Genome Database of Fish
T-Coffee: Tree based Consistency Objective Function For alignment Evaluation
F-primer: Forward primer
R-primer: Reverse primer
SSGSP: Single-Species Group Single Primer Pairs
MSGSP: Multispecies Group Single Primer Pairs
MSGMP: Multispecies Group Multiple Primer Pairs
EP: Exclusive Primer Pairs
NEP: Non-exclusive Primer Pairs
SP: Single primer pairs
MP: Multiple primer pairs
SSG: Single-species group
MSG: Multispecies Group
MFG: Multi-Family Group

## References

[1] Nelson JS (2006). Fishes of the world.

[2] Fischer J. Fish identification tools for biodiversity and fisheries assessments, Reciew and quidance for decision-marker. FAO Fisheries and Aquaculture Technical Paper (FAO). 2013. p. 585.

[3] Costa FO, Carvalho GR (2007). The Barcode of Life Initiative: synopsis and prospective societal impacts of DNA barcoding of fish. Life Sci Soc Policy.

[4] Hassold S, Lowry PP, Bauert MR (2016). DNA Barcoding of Malagasy rosewoods: towards a molecular identification of CITES-listed Dalbergia species. PLoS ONE.

[5] Candek K, Kuntner M (2015). DNA barcoding gap: reliable species identification over morphological and geographical scales. Mol Ecol Resour.

[6] Sutou M, Kato T, Ito M (2011). Recent discoveries of armyworms in Japan and their species identification using DNA barcoding. Mol Ecol Resour.

[7] Ward RD, Zemlak TS, Innes BH (2005). DNA barcoding Australia's fish species. Philos Trans R Soc Lond B Biol Sci.

[8] Ward RD, Zemlak TS, Innes BH (2005). DNA barcoding Australia's fish species. Philos Trans R Soc B.

[9] Geller J, Meyer C, Parker M, Hawk H (2013). Redesign of PCR primers for mitochondrial cytochromecoxidase subunit I for marine invertebrates and application in all-taxa biotic surveys. Mol Ecol Resour.

[10] Park DS, Suh SJ, Oh HW (2010). Recovery of the mitochondrial COI barcode region in diverse Hexapoda through tRNA-based primers. BMC Genomics.

[11] FAO 2020. The State of World Fisheries and Aquaculture 2020. Sustainability in action. Rome. 10.4060/ca9229en. Accessed 25 Dec 2020.

[12] Betancur-R R, Wiley EO, Arratia G (2017). Phylogenetic classification of bony fishes. BMC Evol Biol.

[13] Iwasaki W, Fukunaga T, Isagozawa R (2013). MitoFish and MitoAnnotator: a mitochondrial genome database of fish with an accurate and automatic annotation pipeline. Mol Biol Evol.

[14] Federhen SJ (2012). The NCBI taxonomy database. Nucleic Acids Res.

[15] Langmead B, Salzberg SL (2012). Fast gapped-read alignment with Bowtie 2. Nat Methods.

[16] Notredame C, Bio DE. Utilisation des Algorithmes Genetiques pour L’analyse de Sequences Biologiques. 1998.

[17] Sievers F, Higgins DG. Clustal Omega, accurate alignment of very large numbers of sequences. In: Multiple sequence alignment methods. Methods in molecular biology (methods and protocols), vol. 1079. Springer; 2014. p. 105–16. 10.1007/978-1-62703-646-7_6 24170397

[18] Rozen S, Skaletsky H (2000). Primer3 on the WWW for general users and for biologist programmers. Methods Mol Biol.

